# The Trend of Dentistry

**Published:** 1915-03

**Authors:** A. E. Webster


### THE TREND OF DENTISTRY.

BY A. E. WEBSTER, M.D., D.D.S., L.D.S.

During the last week of January the National Faculties Association and the American Dental Teachers met at Ann Arbor, Michigan. There could be no better place in America for such a meeting. It is a real eye opener, and mental stimulant and nerve sedative for the bustling professor of the so-called dental department of a university from the large cities to spend a few days in a university town. How comforting it must have been to many to breathe the dustless, crisp air and see the snow lie clear and white upon the streets without any attempt being made to cart it away. And above all, to see real live squirrels playing among the trees and on the streets, and no one desiring to kill them.

Only such surroundings can develop ideals. The dental department is a part of a great university where the professors, mid suitable surroundings, have time to think. Their incomes are secured, their equipment adequate, the student body of the best type. Several of the professors devote their whole time to research and teaching. They each have their own rooms, library and equipment. Their lives are devoted to the students, dentistry, the university and the State. Compare this in results with the professor or teacher who rushes into the college worn out from other duties and delivers a lecture or makes a demonstration and is gone for a week. He doesn't know the students and the students do not know him. The students may by this method learn a *lot of things*, but can never develop true ideals of life.

The dental library and museum of Michigan give an opportunity for teaching true dentistry far beyond what is done with many a brilliant faculty. The good results of the meeting where such opportunities for development abound will be of even greater value to dentistry in America than the papers and discussions at the sessions.

At no other meeting was there so much interest shown in the new books exhibited. Authors and publishers are more and more inclined to discuss making of books with the Text Book Commission.

Methods of teaching come up for review each year with great advantage to all. Each time the subject is discussed some teacher will discard a bad method even if he won't study psychology.

The Faculties Association decided to go to a four year course the autumn of 1917. With the beginning of this term Dr. Black outlined a course which, in his opinion, would develop more of a thinking, without making him less of a *doing* dentist.

Dr. Arthur D. Black discussed how to make the dental student a better dentist student.

Concurrent with the first two years of dental study the student should take further work in literary and scientific work in the regular departments of the university. This would have the advantage of the association with the literary departments, teach the young men to read, study and appreciate English literature without directing their attention away from dentistry or delaying the time from the study or practise of fingercraft. Many of the teachers thought quite favorably of the proposal.

In introducing the subject Dr. Black pointed out that there was a decided change in the demands of the dentist within the past few years, and the colleges must adapt themselves to the new conditions. In the past dentists had recommended young men into dentistry who were not inclined to think or read. Young men themselves chose dentistry because they thought they had some natural aptitude for mechanics and little for study. Such men have filled the ranks of the profession and will continue to do so until a class of men are graduated who have been trained to read, study and think. The public demands today that a dentist shall know all about the diseases of the mouth and jaws and their bearing on general health. The dentist who is only a mechanic is rapidly losing his place and the teachers are preparing to meet the new demands. It was quite clear throughout the whole meeting that the new demands are pressing.—*Editorial in Dominion Journal.*

